# Case report: Chronic granulomatous disease presenting with early-onset inflammatory bowel disease and normal oxidative burst testing

**DOI:** 10.3389/fped.2022.964025

**Published:** 2023-01-11

**Authors:** Sanjib Mondal, Pandiarajan Vignesh, Sathish Kumar Loganathan, Kanika Arora, Jhumki Das, Amit Rawat, Surjit Singh

**Affiliations:** Allergy Immunology Unit, Department of Pediatrics, Advanced Pediatrics Centre, Post Graduate Institute of Medical Education and Research, Chandigarh, India

**Keywords:** chronic granulomatous disease, dihydrorhodamine, nitroblue tetrazolium, colitis, residual NADPH oxidase

## Abstract

**Background:**

Due to the lack of widespread availability of flow cytometry services for immunodeficiency, nitroblue tetrazolium test (NBT) is the commonly used screening modality to identify patients with chronic granulomatous disease (CGD) in developing countries.

**Procedure:**

We report a child with X-linked CGD with residual NADPH oxidase activity who had an indeterminate NBT result even in the presence of classical manifestations of CGD.

**Results:**

A 7-year-old boy presented with recurrent episodes of inflammatory colitis and *Burkholderia cepacia* septicaemia at the age of 3 years. He also had cervical adenitis due to *Mycobacterium tuberculosis*. NBT performed on multiple occasions was not suggestive of CGD. Dihydrorhodamine (DHR) test using phorbol myristate acetate (PMA) as a stimulant revealed a small blunt peak suggestive of AR-CGD; however, significant reduction in NADPH oxidase activity was noted with milder stimulants such as *Escherichia coli* and *Staphylococcus aureus*. Genetic analysis revealed a hemizygous pathogenic variant in *CYBB*. Flow cytometry showed diminished gp91phox expression in the patient's neutrophils suggestive of X-linked CGD.

**Conclusion:**

Our case highlights that early-onset inflammatory bowel disease can be a presenting manifestation of CGD and diagnosis of CGD can be missed if NBT alone is used for screening, especially in the presence of NADPH oxidase activity. Diagnosis of “CGD with residual NADPH oxidase activity” requires a high degree of clinical suspicion, and performing DHR with different stimulants can unravel the diagnosis.

## Highlights

1. Diagnosis of chronic granulomatous disease (CGD) with residual NADPH oxidase activity can be missed if nitroblue tetrazolium test (NBT) is used as the only screening modality.
2. A high index of clinical suspicion and early referral to tertiary-care centers, where facilities for performing dihydrorhodamine (DHR) testing are available, are needed to avoid significant delays in diagnosis.
3. Dihydrorhodamine test with the use of milder neutrophil stimulants such as *Escherichia coli* or *Staphylococcus aureus* is useful to identify CGD with residual NADPH oxidase activity.

## Introduction

Chronic granulomatous disease (CGD) is an inborn error of the NADPH oxidase system characterized by recurrent infections and episodes of hyperinflammation (1). While the X-linked form of CGD is more prevalent in Western countries, autosomal recessive (AR) forms of CGD are common in countries with high rates of consanguinity and endogamous marriage (2). Usually, clinical manifestations in X-linked CGD are more severe when compared with AR-CGD (3). However, in the presence of partial activity of NADPH oxidase, children with X-linked CGD may also present with milder or fewer episodes of infections (4).

Diagnosis of CGD in developing countries such as India is usually based on the nitroblue tetrazolium test (NBT). NBT assay is inexpensive and readily available at most centres. Dihydrorhodamine (DHR) testing by flow cytometry is only available at specialized centers in India, and phorbol myristate acetate (PMA) is usually used as the stimulant for both NBT and DHR tests (5).

Patients with CGD with residual NADPH oxidase activity can sometimes be missed if the DHR assay is performed with only PMA (6).

## Case details

A 7-year-old boy, firstborn of non-consanguineous parents, presented to our institute with recurrent episodes of colitis noted from early infancy. The child underwent colonoscopy five times from different hospitals in the last 6 years and was diagnosed with inflammatory bowel disease. A colonoscopy revealed diffuse erosions, ulcerations, granularity, and loss of vascularity in the rectum, sigmoid colon, and descending colon. In addition, the transverse colon showed discrete erosions. However, ascending colon and terminal ileum were found to be normal. Colonoscopy-guided biopsy showed patchy ulcers with neutrophilic exudate, crypt abscess, and crypt loss. Multiple mucosal granulomas with dense epithelioid cells were noted. He was managed as a case of inflammatory bowel disease with mesalamine and intermittent doses of oral glucocorticoids. He showed clinical response with oral glucocorticoids; however, symptoms recurred once the steroid doses were tapered or stopped. For the same, the child was later initiated on oral mesalamine, which was continued for the last 3 years. He had a past history of *Burkholderia cepacia* septicaemia at 3 years, pneumonia requiring hospital admission at 5 years, and an episode of cervical lymphadenitis at 6 years. He had received one course of antitubercular therapy (ATT) based on empirical grounds. NBT assay was performed on multiple occasions at different facilities; however, there was a reduction of NBT to formazan on every occasion that was not conclusively suggestive of CGD.

**Figure 1:**
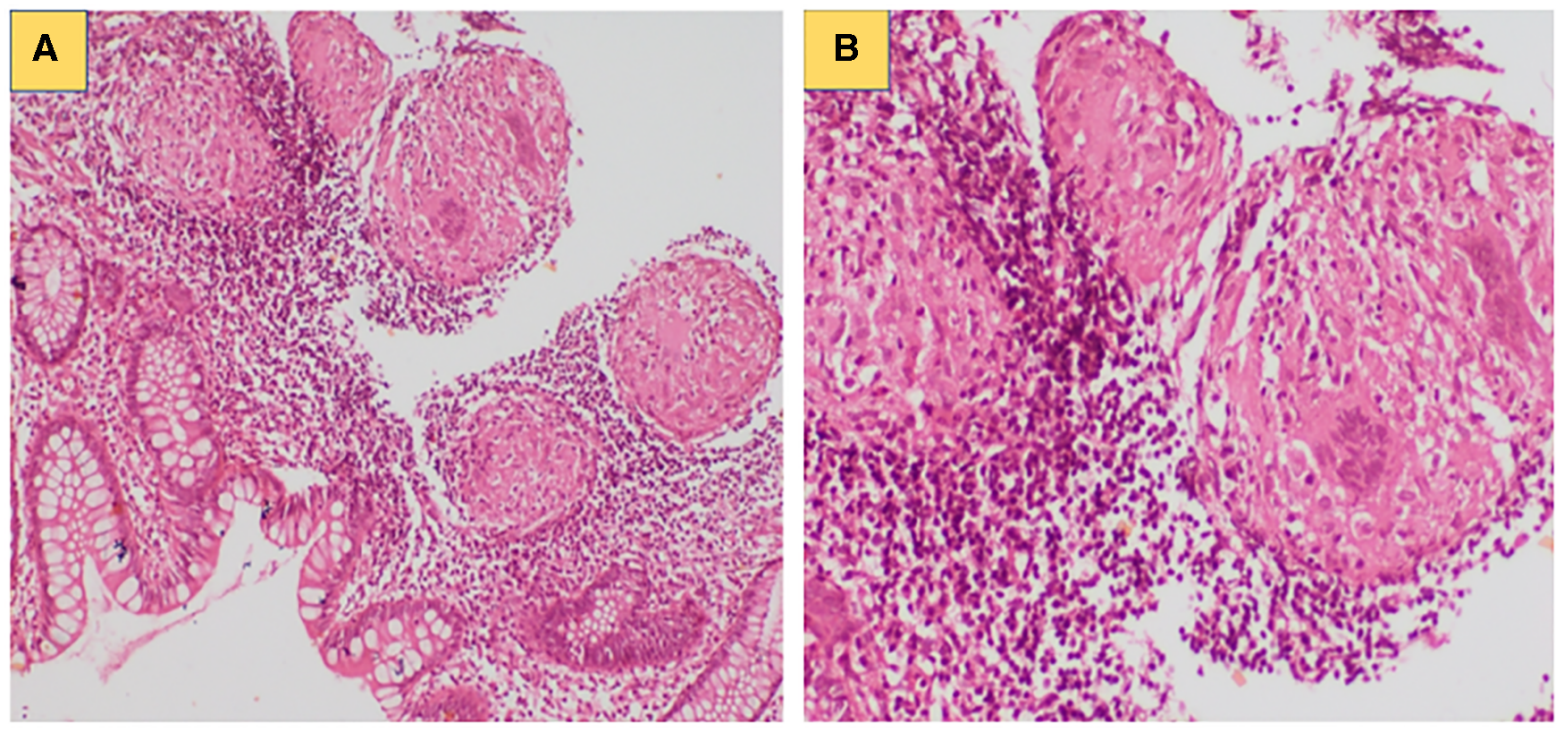
(**A**) Colonic biopsy showing focal excess of mononuclear inflammatory cells with increased number of eosinophils in lamina propria; (**B**) Compact non-caseating granuloma noted in the colonic submucosa.

He had an average build and a healthy scar the bacille Calmitte-Guerin (BCG) vaccination site. He had moderate pallor and left anterior lower cervical lymphadenopathy. The rest of the clinical examination was unremarkable. Complete blood count showed anaemia (Hb 7 g/L), neutrophilic leukocytosis (total leucocyte count 14.1 × 10^9^/L; polymorphs 66%, lymphocytes 26%, monocyte 7%, and eosinophil 1%), and thrombocytosis (platelet 498 × 10^9^/L). Erythrocyte sedimentation rate was 70 mm at the first hour. C-reactive protein was 66 mg/L. Fine needle aspiration cytology from the cervical lymph node showed scattered multinucleated giant cells along with few immunoblasts, plasma cells, and histiocytes. Cartridge-based nucleic acid amplification test (CBNAAT) from lymph node aspirate revealed *Mycobacterium tuberculosis* complex. Endoscopic biopsy from the sigmoid colon revealed features suggestive of inflammatory colitis (Figure 1).

## Immunological tests

Immunological work-up showed a normal proportion of B, T, and NK cells and high IgG levels [IgG 27.6 g/L (normal 4.4–11.0 g/L), IgM 2.08 g/L (normal 0.4–2.4 g/L), and IgA 3.41 g/L (normal 0.5–1.8 g/L)]. Serology for human immune deficiency virus serology was non-reactive.

**Figure 2:**
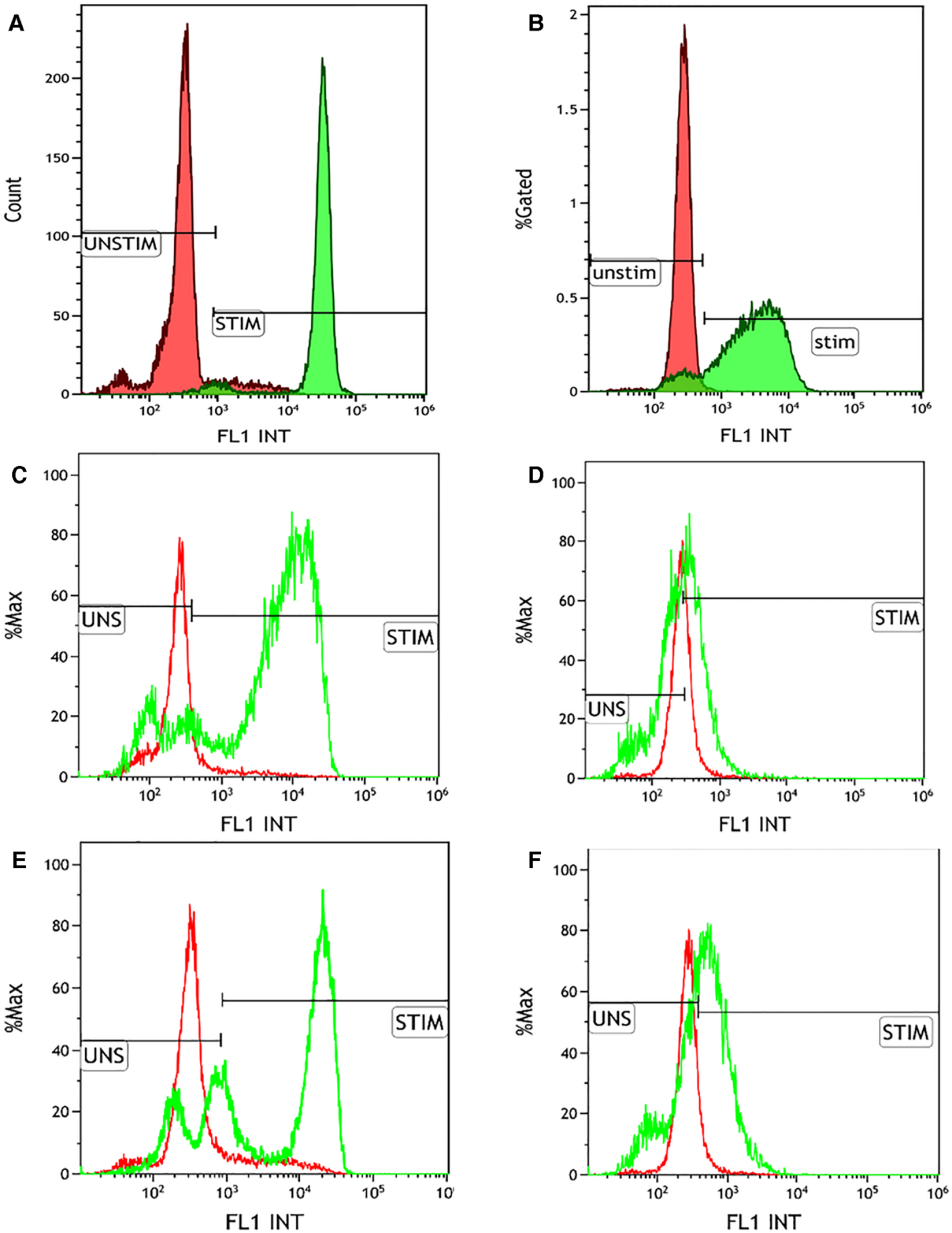
Dihydrorhodamine (DHR) test using multiple stimulants. (**A**) Healthy control showing normal right shift of the neutrophils stimulated by phorbol myristate acetate (100 ng/mL) (MFI of unstimulated and stimulated neutrophils is 319.68 and 32605.18, respectively; S.I. of 101.99; % positivity of the stimulated neutrophils 97.16%); (**B**) DHR plot of the patient showing stunted broad-based peak of the PMA-stimulated neutrophils (MFI of unstimulated and stimulated neutrophils is 272.29 and 3116.69, respectively; S.I. of 11.44; % positivity of the stimulated neutrophils 88.7%); (**C**) Healthy control showing normal right shift of the neutrophils stimulated by *Escherichia coli* (OD600-0.1) (MFI of unstimulated and stimulated neutrophils is 299.9 and 27464.1, respectively; S.I. of 91.57; % positivity of the stimulated neutrophils 87.44%); (**D**) DHR plot of the patient showing absent right shift of the *E. coli* stimulated neutrophils (MFI of unstimulated and stimulated neutrophils is 245.57 and 477.52, respectively; S.I. of 1.94; % positivity of the stimulated neutrophils 13.67%); (**E**) Healthy control showing normal right shift of the neutrophils stimulated by *Staphylococcus aureus* (OD600-0.1) (MFI of unstimulated and stimulated neutrophils is 299.9 and 20631.91, respectively; S.I. of 68.79; % positivity of the stimulated neutrophils 69.7%); (**F**) DHR plot of the patient showing absent right shift of the *S. aureus* stimulated neutrophils (MFI of unstimulated and stimulated neutrophils is 245.57 and 987.59, respectively; S.I. of 4.02; % positivity of the stimulated neutrophils 36.57%).

**Figure 3:**
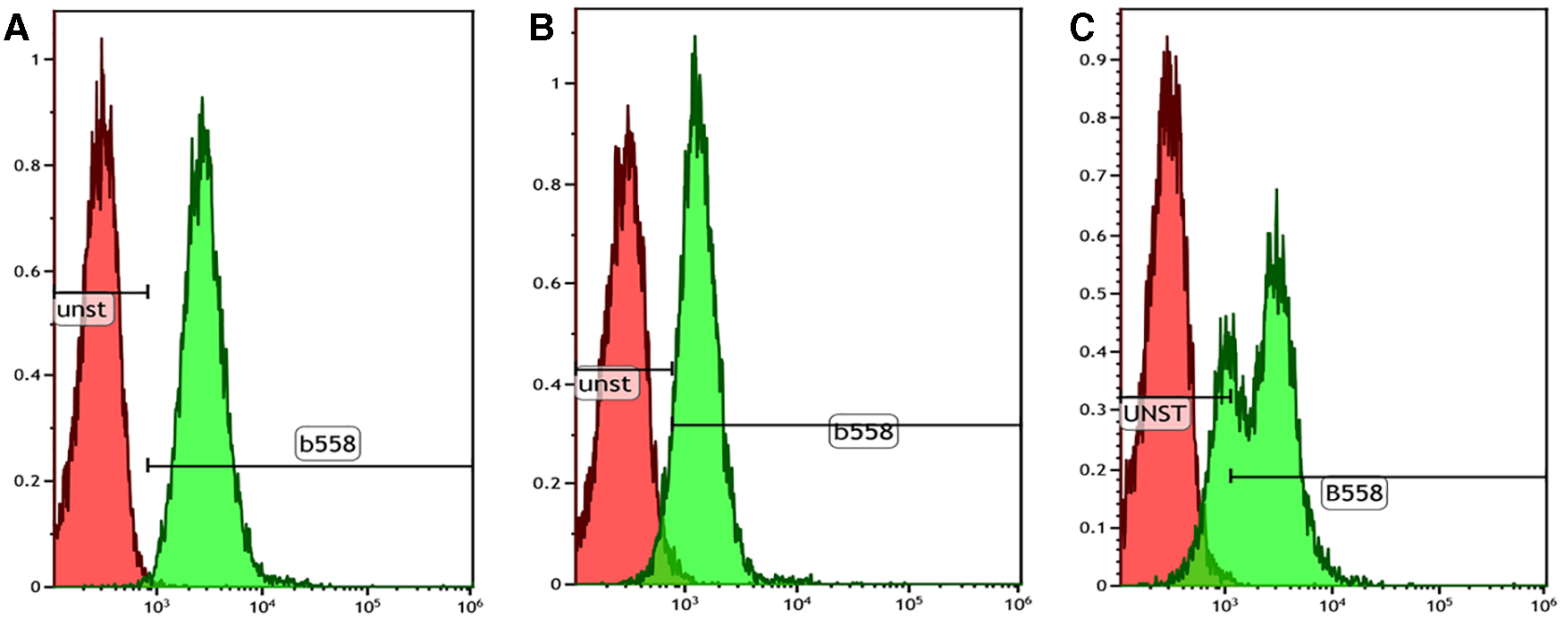
Cytochrome B558 expression in neutrophils by flow cytometry. (**A**) Healthy control showing normal B558 expression in the stained neutrophils (99.8% with stain index 9.7); (**B**) Index patient showing mildly reduced B558 expression in the stained neutrophils (71.3% with stain index 4.5); (**C**) Mother showing bimodal pattern of B558 expression suggestive of a carrier state of X-linked CGD.

NBT assay showed a reduction in 60% of neutrophils in the patient compared with the 90% reduction in the healthy control, which was not conclusive of CGD. This prompted us to do further investigations, such as DHR and genetic analysis. Flow cytometry-based DHR test using PMA (100 ng/mL) as a stimulant showed a broad-based short-peak fluorescence of stimulated neutrophils (Figures 2A,B). However, the percentage of NADPH oxidase positivity was almost comparable (88%) with healthy age-matched control (97%). We subsequently performed the DHR test using milder stimulants such as *Staphylococcus aureus* (OD600-0.1) (Figures 2C,D) and *Escherichia coli* (OD600-0.1) (Figures 2E,F). A significant reduction of NADPH oxidase positivity was noted with these stimulants. B558 expression in neutrophils in the child showed a reduced expression (Figure 3B). B558 expression of the mother showed double peak fluorescence suggestive of carrier state for X-linked CGD (Figure 3C). His mother had no history of fatigue or infection. In addition, she had no features suggestive of autoimmunity.

## Molecular assays

Next-generation sequencing using a targeted gene panel consisting of 44 common PID genes (including 5 genes implicated in CGD) revealed a hemizygous missense variant (c.925G > A, p.Glu309Lys) in exon 9 of *CYBB*. The variant detected was confirmed by Sanger sequencing. His mother was found to be a heterozygous carrier for the same variant.

## Patient follow-up details

The index patient was commenced on ATT (isoniazid, rifampicin, ethambutol, and pyrazinamide), itraconazole and cotrimoxazole prophylaxis. He was also started on sulfasalazine (30 mg/kg) along with oral prednisolone (1 mg/kg) for the management of colitis. Oral prednisolone doses were tapered and stopped over the next 3 months. He received ATT for a 1-year duration. The patient has remained well on follow-up at 1.5 years. Parents have been counselled for haematopoietic stem cell transplantation, and Human Leukocyte Antigen (HLA) typing of the patient and family members is planned.

## Discussion

Clinical suspicion is the first and most essential step for diagnosing CGD. Our child had classical clinical symptoms suggestive of CGD—early-onset inflammatory colitis, pneumonia, and history of septicaemia due to a signature organism (viz., *Burkholderia cepacia*) and mycobacterial lymphadenitis. However, the diagnosis of CGD could not be established using NBT assay alone. This has led to a significant delay in establishing the correct diagnosis. Early clinical diagnosis of CGD with residual NADPH oxidase activity requires a high index of suspicion, and the diagnosis may be delayed if only NBT is performed. Furthermore, abnormal but not conclusive results should be followed up with further investigations.

Vowells et al. described different characteristic patterns of DHR fluorescence in X-linked CGD, AR-CGD, and X-linked carriers of CGD. Neutrophil oxidative index less than 2 in DHR is classically seen in patients with X-linked CGD (7). However, our child showed short, broad peak fluorescence in the DHR assay, and the neutrophil oxidative index was suggestive of residual NADPH oxidase activity, which is usually seen in AR-CGD due to p47phox defect. A short, broad peak detected on DHR assay is widely considered pathognomonic of AR-CGD with p47phox defect. As in the present case, a broad peak may also be seen in cases with residual oxidase activity due to other genetic defects. Milder stimulants like formyl-methylene-leucyl-phenylalanine (fMLP) are used in DHR to uncover the diagnosis of CGD (6). In our case, we performed DHR with milder stimulants *S. aureus* (OD600-0.1) and *E. coli* (OD600-0.1), which revealed a reduction in NADPH oxidase activity.

Hemizygous mutation in *CYBB* leads to defective gp91phox component of NADPH oxidase complex. The defect has been described previously in three forms—(1) total absence of gp91phox with the absent activity of NADPH oxidase (gp91phox^−^), (2) partial expression of gp91phox with residual activity of NADPH oxidase (gp91phox^+^), and (3) normal expression of gp91phox with nonfunctional NADPH oxidase (gp91phox^0^) (4). In our child, the residual activity of NADPH oxidase was noted in the DHR assay. However, a significant reduction was noted only with milder stimulants (*S. aureus* and *E. coli*). Patients with p40phox defect, a form of AR-CGD, can have a normal DHR assay when the test is performed only with PMA as a stimulant. These patients clinically present only with minimally invasive infections and features of colitis (8). A final genetic diagnosis is required for prognosis, counseling, and family screening (9). The child had a missense variant in exon 9 of *CYBB* that has been previously reported to be associated with residual NADPH oxidase activity (4). These patients with residual NADPH oxidase activity may present with less severe clinical manifestations when compared with usual X-linked forms of CGD.

NBT is a valuable screening test for diagnosing CGD, especially in resource-limited countries. In India, most centers use NBT for the diagnosis of CGD. In a few instances, DHR testing by flow cytometry would be necessary to establish the diagnosis of CGD where results of NBT are equivocal or negative, especially in cases of CGD with residual NADPH oxidase activity. However, only a few centers have facilities for DHR testing in India. Most of the centers use PMA as a stimulant in DHR testing (5). In some instances, DHR assay with conventional PMA stimulation can miss the diagnosis of CGD with residual NADPH oxidase activity. In such scenarios, performing DHR with milder stimulants is required to confirm the diagnosis.

Therefore, a high suspicion is required for early diagnosis of CGD, especially in the presence of residual NADPH oxidase activity. NBT alone can miss the diagnosis of CGD in such instances. These cases would need DHR testing with multiple stimulants to establish the diagnosis. Also, such patients would benefit from early referral to tertiary-care centers where clinical and laboratory immunology expertise are available. Our report also calls for increasing awareness of CGD among physicians and the need to expand existing clinical immunology services in developing countries.

## Data Availability

The raw data supporting the conclusions of this article will be made available by the authors, without undue reservation.
